# Peculiar Gin Accident

**Published:** 1905-12

**Authors:** J. P. Oliver

**Affiliations:** Caldwell, Texas


For Texas Medical Journal.

### Peculiar Gin Accident.

BY J. P. OLIVER, M. D., CALDWELL, TEXAS.

The case before you today, gentlemen, for consideration is perhaps without a parallel in the history of traumatic accidents, at least so far as the writer's data extends. This young man presents a very fair specimen of physical manhood, although there is some hereditary diathesis in the family history. He was a pressman at the gin of the American Round-bale Cotton Company at Caldwell, Texas, and on the 15th day of September last about 2 p. m., was so unfortunate as to have his left hand caught in the press while attempting to remedy some defect in the position of the bagging. On arrival, I suppose not over thirty or forty minutes after the accident, the young man was sitting against the wall of the house on the ground as pale as death, seemingly very much shocked, with the skin of his arm and hand hanging by his side very much like an empty shirt sleeve.

After administering some stimulants and anodynes hypodermically, he was removed to the residence of T. F. Gidies, near by, where he was made as comfortable as his condition would permit, and the arm was prepared for dressing. The peculiar wound can not be satisfactorily explained upon any hypothesis other than that there were two antagonistic forces acting, one on the part of the press, the other of the young man, the arm being the leverage, these forces acting so forcefully that the skin gave way in the armpit and around the upper part of the humerus; in fact, the skin of the whole arm and hand were stripped off very much like the skinning of a rabbit or squirrel. It is perhaps due Mr.

---

*Read before the Brazos Valley Medical Association, at Caldwell. Texas May 11, 1904.

Gillie and his wife to say that but for the kindness shown this young man for six weeks or more, the case might have terminated differently.

Assisted by Dr. Matthews, it was thought best to try and save the arm, as the main blood vessels and nerves were intact. We knew the hand would be lost, as the lacerations were so severe as to destroy the muscles, blood vessels and nerves. The fingers were split and the hand brought down; the skin readjusted as best we could, and sutured at the original rent. Absorbent cotton was placed all over the arm and hand and antiseptic roller bandages applied.

The patient reacted fairly well in ten or twelve hours, but with reaction came high temperature, and for eight or ten days death seemed inevitable. Consultation was held, the result of which seemed to be that it would not avail any hope of saving the life of the patient. My opinion, however, was to amputate at the shoulder joint and give him the benefit of the doubt of its availability. My reasons for the joint operation was the possibility of getting sound flesh for stump. The amputation was performed at the upper third ten days after the accident. Dr. Matthews gave the anesthetic and Dr. Kruger assisted in the amputation. The patient bore the operation nicely, and reacted very well in a short while. All things considered, we thought the patient had a fighting chance for his life. The outer part of the wound healed by first intention; two supra clavicular abscesses formed and were hard to control, but by active antiseptic treatment the infection was finally subdued.

The details of the technique of this case has purposely been omitted, as it would be too voluminous; suffice to say that strict asepsis and antisepsis was observed all the while he has been under my observation. It is regretable that my data of grafting were misplaced or lost, so that I can not give the general detail of procedure. I will state, however, that 104 grafts have been applied to both wounds, 8 on his right hand and 96 to the stub of the arm,—62 from his mother, who was a very fine subject, there being no constitutional vice; 4 from the belly of a puppy three or four weeks old; the balance from friends, promiscuously, which I think is not a good idea. I believe, for successful grafting, that the party supplying the grafts should have no constitutional vices, and very strict asepsis should be observed all the time of grafting. Dr. Kruger assisted me in applying the first grafts.

During the Houston meeting in January last, this case was incidentally mentioned and discussed to some extent, at which time the wound was progressing nicely, and most of the grafting had been successful. But on the 18th of January a public salesday was had in town, and this young man came to the sale and remained all day. In a few days the wound became reinfected and many of the grafts broke down and suppurated profusely, which required several days to subdue.

I can not dismiss this case, gentlemen, without referring to a mother's love for her son, whose fidelity never faltered, no matter on what occasion, even to the giving of sixty-odd grafts off of her own for the sake of her boy's life. His only sister was, nevertheless, attentive ever since the 15th of September, 1903; both have moved him day and night; they have ministered to his every want. What wondrous love a mother has for her child none but an All-wise God can tell.

One other point and I have done. This young man's father died of some form of cancer of the face. His elder brother is an asthmatic, and when a child had an inveterate herpetic eruption for several years. Tuberculosis is hereditary in the family. May not these diatheses have exercised an influence in protracting this case? His weight has decreased some twelve or fifteen pounds in the last few months.

